# Postpartum anemia and its determinant factors among postnatal women in two selected health institutes in Gondar, Northwest Ethiopia: A facility-based, cross-sectional study

**DOI:** 10.3389/fmed.2023.1105307

**Published:** 2023-04-20

**Authors:** Getachew Mesfin Bambo, Samuel Sahile Kebede, Chomaw Sitotaw, Elias Shiferaw, Mulugeta Melku

**Affiliations:** ^1^Department of Hematology and Immunohematology, School of Biomedical and Laboratory Sciences, University of Gondar, Gondar, Ethiopia; ^2^Department of Medical Laboratory Science, College of Health Sciences, Mizan-Tepi University, Mizan, Ethiopia; ^3^University of Gondar Comprehensive Specialized Hospital Laboratory, Gondar, Ethiopia; ^4^College of Medicine and Public Health, Flinders University, Adelaide, SA, Australia

**Keywords:** determinant factors, postpartum anemia, postnatal women, Ethiopia, postpartum hemarrhage

## Abstract

**Background:**

Anemia is highly prevalent globally and disproportionately affects postnatal women. It is a significant cause of maternal mortality and morbidity globally.

**Objective:**

The main aim of this study was to determine the extent of postpartum anemia and associated factors among postnatal women in two selected health facilities in Gondar, Northwest Ethiopia.

**Methods:**

A facility-based, cross-sectional study was conducted among 282 postnatal women from March to May 2021. A systematic sampling technique was used to recruit study participants from each institute. Sociodemographic, obstetric, and clinical data were collected through a semi-structured questionnaire. A venous blood sample was collected to determine the red blood cell parameters. A thin blood smear preparation was performed to examine blood morphology. In addition, direct wet mount and formalin-ether sedimentation techniques were used for stool examination to identify intestinal parasites. Data were entered into EpiData and exported to Stata 14 for statistical analysis. Descriptive statistics were presented in text, tables, and figures. A binary logistic regression model was used to identify factors associated with postpartum anemia. A *p*-value <0.05 was considered statistically significant.

**Results:**

The proportion of postpartum anemia was 47.16%; 95% CI; 41.30–53.03 with moderate, mild, and severe anemia accounting for 45.11, 42.86, and 12.03%, respectively. The majority of the anemia (94%) was of the normocytic normochromic type. It was associated with postpartum hemorrhage (AOR = 2.23; 95% CI: 1.24–4.01), cesarean section (AOR = 4.10; 95% CI: 2.11–7.78), lack of iron and folate supplementation during pregnancy (AOR = 2.12; 95% CI: 1.17–4.02), and low diet diversity level (AOR = 1.83; 95% CI: 1.05–3.18).

**Conclusion:**

The prevalence of anemia was found to be a major public health concern. Iron and folate supplementation during pregnancy, improved management of PPH, an effective cesarean section with post-operative care, and taking a diversified diet will reduce the burden. Therefore, identified factors should be considered to prevent and control postpartum anemia.

## Introduction

Anemia is defined as a decrease in the mass of red blood cells (RBCs) or a low level of hemoglobin (Hb) relative to the normal reference range. The World Health Organization (WHO) defines it as Hb < 12.0 gm/dl in adult, non-pregnant women. However, there is no well-defined agreement for postpartum anemia (PPA), nonetheless, which is still defined as Hb < 12 gm/dl (1, 2). The postpartum period is defined as the first 6 weeks following childbirth (3).

Anemia is also defined as tissue hypoxia due to inadequate oxygen delivery in the tissue. The pathophysiology of anemia is multi-factorial and complex, which might be associated with a predisposition in genes for Hb, enzymatic deficiencies, chronic and acute blood loss, nutrient deficiencies, infection, hemorrhage, chronic disease, bone marrow disorders, and other factors. This may be due to inadequate or defective erythropoiesis from food shortages, bone marrow infiltration, inflammation, or hereditary Hb disorders and severe erythrocyte loss related to hemolysis and acute blood bleeding (4, 5).

Indeed, iron deficiency is the most common micronutrient deficiency and the most common cause of anemia in the world. About half (50%) of the cases of anemia are attributed to iron deficiency. Iron deficiency anemia (IDA) is more prevalent in low-income countries (6). Anemia is a serious problem and challenge in the world that can affect about one-third of people around the world (women, young children, and individuals with long-term diseases) in both developed and developing countries (7, 8). It is a fact that anemia is more prevalent among children and women of reproductive age, including postnatal women, and can cause morbidity and mortality in women in addition to fetal consequences (9).

Anemia is a significant cause of maternal mortality and morbidity on a global scale. It affects 38% of pregnant women and 29% of non-pregnant women, with the highest proportion in central and west Africa (56%) (10). Around 500 million women of reproductive age are affected by anemia, which is a major public health challenge for low- and middle-income countries (11). One of the developing countries, Ethiopia, had the highest rates of maternal deaths in 2016, with 412 women dying for every 10,000 live births (12). This mortality is mainly associated with prolonged iron loss due to postpartum hemorrhage (PPH), which is a leading predictor of cardiac arrhythmias (13, 14). It is also associated with lower global household income, cognitive and psychological impairment, poor quality of life, emotional instability, and postpartum depression (1, 15).

In addition, PPA has the highest risk of endometritis and thrombotic complications secondary to iron deficiency and local ischemia (16, 17). Furthermore, the prevalence of anemia among lactating women has increased from 23.03 to 28.3% in Ethiopia over the past two and a half decades (2011–2016) (18). PPA is primarily linked to poor economic development, obstetric problems, and low nutritional status. Evidence suggests that obstetric factors are a significant source of PPA, leading to morbidity and mortality in women of reproductive age (19). During delivery, women experienced traumatic processes, leading to PPH. Different studies investigated the risk factors for PPA, including PPH, maternal residence, blood loss, and maternal level of education. This could be attributed to active bleeding during delivery, which causes a drop in Hb levels both before and after delivery (20).

It is also characterized by their socioeconomic context. Higher prevalence rates (57.2%) of anemia among rural women aged 20 years and older were observed in India (21). PPA is also a common problem in countries with stronger economies (22). Low maternal education levels, poor socioeconomic status, and living in rural areas all play a significant role in the development of anemia in women (23). Women with low dietary diversity had a higher risk of anemia (24). During pregnancy, improved and increased adherence to iron and folic acid was found to reduce the possible risk of anemia in postnatal women. Vitamin and mineral deficiencies may worsen during pregnancy due to increased energy and nutrient demands, causing adverse outcomes for both mother and child (25).

In addition to this, anemia in women of reproductive age is a significant issue, and the WHO has set a global goal of attaining a 50% reduction in anemia in women of reproductive age by 2025 (26). To achieve the WHO target, addressing anemia in postnatal women is of great importance. However, there are only a few rigorous studies conducted on PPA in Africa, particularly in Ethiopia. Although blood morphology is helpful in the diagnosis and categorization of the pathophysiology of anemia (27). In addition, parasitic infection is an independent predictor of anemia. However, the available studies did not reveal this. As a result, this study highlighted important findings that can help improve the maternal healthcare system.

## Methods

### Study area, design, and period

A facility-based cross-sectional study was conducted in the town of Gondar, in the Amhara regional state, from March to May 2021. Gondar has one hospital and eight public health centers. The estimated population projection for Gondar was 362,000 in 2020 (28). Gondar is situated about 727 kilometers (km) northwest of Addis Ababa and 185 km from the city of Bahir Dar. It is located at an altitude of 2,133 meters above sea level at 12° 36′ north and 37° 28′ east, latitude and longitude, respectively (29).

### Population

Source and study population: All postnatal women in Gondar were considered as the source population. All postnatal women who gave birth and attended the postnatal care (PNC) clinic at the Gondar Health Center and the University of Gondar Comprehensive Specialized Hospital (UGCSH) and were available during the data collection period were considered as the study population.

### Sample size determination

The sample size was determined by a single population proportion formula, considering a 5% margin of error at 95% CI, and by taking the 24.3% prevalence reported in the previous study (30):


$$N={\left(Z\alpha /2\right)}^{2}\left(\frac{p\;\left(1-p\right)}{d^{2}}\right)$$

where N = the desired sample size, z = 1.96, standard normal distribution value at 95% CI corresponding to a significant level of alpha, and d = acceptable margin of error.


$$N={\left(1.96\right)}^{2}\left(\frac{0.243\left(0.76\right)}{(0.05^{)2}}\right)=282$$

The total sample size calculated for this study was 282 postnatal women.

### Sampling technique

**Figure 1:**
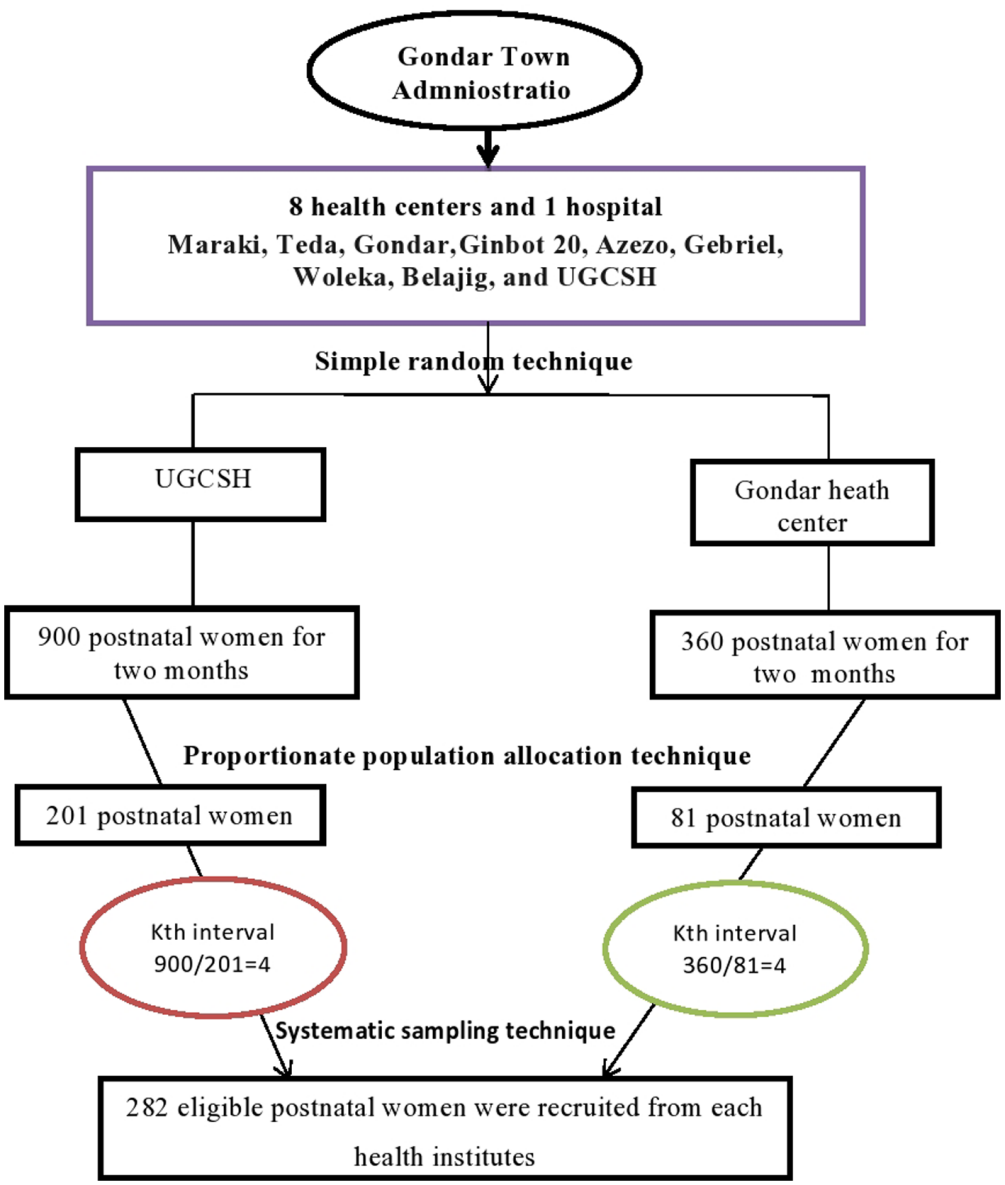
Schematic presentation of sampling technique.

Of the nine public health facilities, two of them, the Gondar Health Center and the UGCSH, were selected using a simple random sampling technique. Using the monthly postnatal women numbers from both facilities (450 from UGCSH and 180 from the Gondar Health Center), the interval value was calculated by dividing the estimated total number of women within the calculated sample size (1890/282 = 6.7) by the proportionate population size in each health facility. For instance, the estimated total number of women in UGCSH during the study period (900) was divided by the interval (4.46) to yield a proportionate sample, which was approximately 201, and the same method was used for the Gondar Health Center, where 81 postnatal women were recruited. Then, a systematic sampling technique was employed, using an interval of four based on the corresponding sample size from each health facility. The lottery method was applied to select the first four participants, and every four women were interviewed until the allocated sample size was achieved (Figure 1).

Inclusion criteria and exclusion criteria: All postnatal women who delivered in the selected health institutions and volunteered to participate were included in the study. Women with a medical history of hypertension, kidney disease, human immunodeficiency virus (HIV), or hepatitis B, too ill to give informed consent, receiving any form of hematinic, or those who had been recently transfused were excluded from the study.

### Variables

Dependent variables: Anemia was a dependent variable. Before determining whether a woman was anemic or not, altitude was considered. The adjustment for altitude was made to account for the decrease in blood oxygen saturation. The Hb level was adjusted using the following formula: (adjustment = −0.032 (altitude _*_ 0.0032808) + 0.022 (altitude _*_ 0.0032808)^2^ and adjHb = Hb – adjustment (when adjustment >0)) (31).

Independent variables: Socio-demographic variables (maternal age, educational level, residence, marital status, religion, family size, occupation), obstetric variables (parity, frequency of antenatal care (ANC) visits, history of abortion and malaria infection, place and mode of delivery, iron and folate supplementation during pregnancy, PPH, intestinal parasites (IP), family planning, birth within 5 years), and nutritional variables such as dietary diversity, weekly meat consumption, daily fruit and vegetable consumption, coffee and tea drinking habits were considered as independent variables.

### Operational definitions

High dietary diversity: Consumption of at least 7 foods from 10 food groups (32, 33).

Low dietary diversity: Consumption of four or fewer foods from 10 food groups (32, 33).

Minimal dietary diversity: Consumption of at least five foods from 10 food groups (33, 34).

Postpartum anemia: Adjusted Hb levels of postnatal women below 11 gm/dl within 24 h and 12gm/dl up to 6 weeks after the birth period result in postpartum anemia, which is further classified as mild (adjHb = 11–11.9 gm/dl), moderate (adjHb = 8–10.9gm/dl) and severe (adjHb <8 gm/dl) (1, 2).

Postpartum hemorrhage: Bleeding during delivery is classified as mild (500-1,000 ml), moderate (1000–2000 ml), and heavy (>2000 ml) postpartum hemorrhage.

### Data collection techniques

After obtaining written informed consent, socio-demographic variables such as age, educational attainment, marital status, occupation, and related characteristics of postnatal women were collected through face-to-face interviews using a pre-tested, semi-structured questionnaire at the obstetric and gynecological wards and PNC units of UGCSH and the Gondar Health Center. Obstetric and clinical data were collected using physical examinations, chart reviews, and laboratory tests. Detailed information on the history of abortion and malaria infection, and iron and folic acid supplements during pregnancy, were collected from the charts. According to the guidelines of the Food and Agriculture Organization (FAO), a Dietary Diversity Score (DDS) was assessed.

Blood sample collection and analysis: A 5 ml venous blood sample was collected by well-trained laboratory personnel using standard aseptic procedures and sent to the hematology laboratory. The blood sample was placed in a labeled tri-potassium ethylenediaminetetraacetic acid (K3-EDTA) tube. An automated, compact 5-part differential hematology analyzer (Beckman Coulter UniCel DxH 800) was used to determine hematological parameters. A drop of blood from a confirmed anemic woman was put on a microscopic slide, and a thin smear was prepared. The smear was stained with undiluted Wright’s staining solution based on standard operating procedures. It was then air-dried, labeled with an identification number, and examined under a microscope with a 100X oil immersion objective by well-trained hematologists. RBC shape, size, color, and cellular inclusions were carefully evaluated for morphologic examination.

Stool sample collection and examination: Pea-sized stool samples were collected from each study participant using a clean, wide-mouth, leak-proof stool cup. Wet mount preparation and the formol-water concentration technique were used to detect IP. Fresh stool samples were mounted on a microscope slide with a wooden applicator stick and emulsified with a drop of physiological saline (0.85% w/v NaCl). The concentration technique was used on the remaining samples for the concentration of IP.

The fecal sample was emulsified in 10 ml of formol-water (10% v/v) in a screw-cap bottle. The suspension was strained into a conical tube by using gauze to extract large fecal particles and centrifuged at 1500 rpm for 3 min. Then, it was re-suspended by using 4–3 ml of formol water and 3–4 ml of ether or ethyl acetate and centrifuged at 3,000 rpm for 1 min. Finally, the supernatant was removed, and the sediment was examined under the microscope. A stool examination was performed according to standard operating procedures. Any helminth eggs, larvae, or cyst stages were detected. All samples were collected through laboratory tests using the participants’ laboratory information checklist.

Data quality management: The questionnaire was prepared in English, translated into the local language (Amharic), and then translated back into English for consistency. Prior to data collection, the data collection tool was pretested on 14 volunteer postnatal women at UGSCH. There were inaccuracies in the measurement of body mass index (BMI), which was therefore omitted. Prior to data collection, orientation was provided to data collectors. During the data collection process, there was close monitoring to ensure data accuracy and consistency. Following blood collection, to avoid hemolysis, the blood was applied to the wall of the test tube. Data were collected by well-trained health professionals (five midwives and three laboratory staff).

Prior to running the participants’ sample, the accuracy of the hematology analyzer was checked with high-, normal-, and low-quality control (QC) materials. All reagents were checked for expiration dates and prepared according to the manufacturer’s instructions. A morphological examination was carried out on the known mean corpuscular volume (MCV) and mean corpuscular Hb concentration (MCHC) of healthy individuals to ensure the quality of the staining reagent. The stool wet mount and suspension were made within the appropriate concentration of sample and reagents. Fresh stool samples were observed within 30 min of collection. Finally, all results were documented and registered with the correct value and units.

### Data processing, analysis, and interpretation

The data were checked for completeness and consistency. Then, they were coded and double-entered into EpiData, version 4.6.0.0, before being imported into Stata, version 14.0, for analysis. The data was cleaned, and preliminary analyses were carried out. Descriptive statistics were summarized using frequency, percentage, median, mean, interquartile range (IQR), and standard deviation for presentation in text, tables, and figures. A binary logistic regression model was used to assess factors associated with the outcome variable. Variables with a *p*-value of ≤0.25 were adjusted for multivariable analysis. In the model, a backward variable selection was used. The *Hosmer* and *Lemeshow* post-estimation statistical test was performed to check the goodness of fit. A *p*-value ≤0.05 was considered statistically significant.

## Results

### Socio-demographic characteristics

A total of 282 postpartum women were included in this study. The median age of the participants was 28 years, with an IQR of 24–30. Approximately 62.8% of the participants were between the ages of 25 and 34. Three-fourths (75.9%) of the respondents were from urban areas. One-third (29.4%) of respondents were illiterate. Of the total participants, 242 (85.8%) were married (Table 1).

**Table 1:** Socio-demographic characteristics of post-natal women in Gondar town, Northwest Ethiopia, 2021 (*n* = 282).

Variables	Categories	*n*	%
Age	15–24	81	28.72
	25–34	177	62.77
	35–49	24	8.51
Maternal educational status	Unable to read and write	83	29.43
	Primary school	70	24.82
	Secondary school	55	19.50
	College and above	74	26.24
Maternal residence	Urban	214	75.89
	Rural	68	24.11
Maternal occupation	Farmer	48	17.02
	Private	72	25.54
	Governmental employee	47	16.67
	Others	115	40.78
Maternal family size	≤4	183	64.89
	5–8	99	35.11
Maternal marriage status	Single	18	6.38
	Married	242	85.82
	Divorced	13	4.61
	Widowed	9	3.20
Maternal religion	Orthodox	244	86.52
	Protestant	11	3.90
	Muslim	27	9.57

Others for maternal occupation represent students, unemployed, merchants, housewives, daily laborers and non-governmental organization workers *n*: represents for frequency and % indicates percentage.

### Obstetric and clinically related characteristics

Of the total study population, 162 (57.5%) women were multiparous. A total of 159 (56.4%) of the participants had experienced PPH. The majority of women, 256 (90.8%) individuals, had at least one ANC visit during pregnancy, with 62.9% of them having at least 4. Most (98.2%) of the women had delivered at a health facility. A total of 209 (74.1%) had a vaginal delivery (Table 2).

**Table 2:** Obstetric and clinical characteristics of postnatal women in Gondar town, Northwest Ethiopia, 2021 (*n* = 282).

Variables	Categories	*n*	%
Parity	Primipara	120	42.55
	Para 2–4	147	52.13
	Grand multiparous	15	5.32
PPH	Yes	159	56.38
	No	123	43.62
Severity of PPH	Mild	70	24.82
	Moderate	62	21.99
	Severe	27	9.57
Place of delivery	At home	5	1.77
	Health institution	277	98.23
Mode of delivery	Vaginal	209	74.11
	CS	73	25.89
ANC follow up	Yes	256	90.78
	No	26	9.22
Frequency of ANC visit	<4 times	95	37.21
	≥4 times	161	62.89
Family planning	Yes	234	82.98
	No	48	17.02
Birth within 5 years	Yes	110	39.01
	No	172	60.99
History of recent abortion	Yes	39	13.83
	No	243	86.17
History of recent malarial infection	Yes	52	18.44
	No	230	81.56
Iron and folate supplementation	Yes	208	73.76
	No	74	26.24

ANC, Antenatal care; CS, cesarean section. *n* represents for frequency and % indicates percentage.

### Dietary characteristics

More than half of the women (150, or 53.2%) had low dietary diversity. A total of 114 (40.4%) women ate meat at least once per week. More than half of the women (158, or 56%) consumed fruit and vegetables at least once a day. A total of 229 women (81.2%) had the habit of drinking coffee; 183 of them (79.9%) preferred to do so 30 min after food consumption (Table 3).

**Table 3:** Nutritional characteristics of postnatal women in Gondar town, Northwest Ethiopia, 2021 (*n* = 282).

Variables	Categories	*n*	%
Diet diversity level	Low	150	53.19
	Medium	118	41.84
	High	14	4.96
Weakly meat	At least once	114	40.43
consumption	Less than once	168	59.57
Daily fruit and vegetable consumption	At least once	158	56.03
	Less than once	124	43.97
Tea drinking	Yes	191	67.73
	No	91	32.27
Tea taking habit	After 30 min of food	160	83.77
	Immediately after food	31	16.23
Coffee drinking	Yes	229	81.21
	No	53	18.79
Coffee taking habit	After 30 min of food	183	79.91
	Immediately after food	46	20.09

*n*: represents for frequency and % indicates percentage.

### Laboratory findings

**Figure 2:**
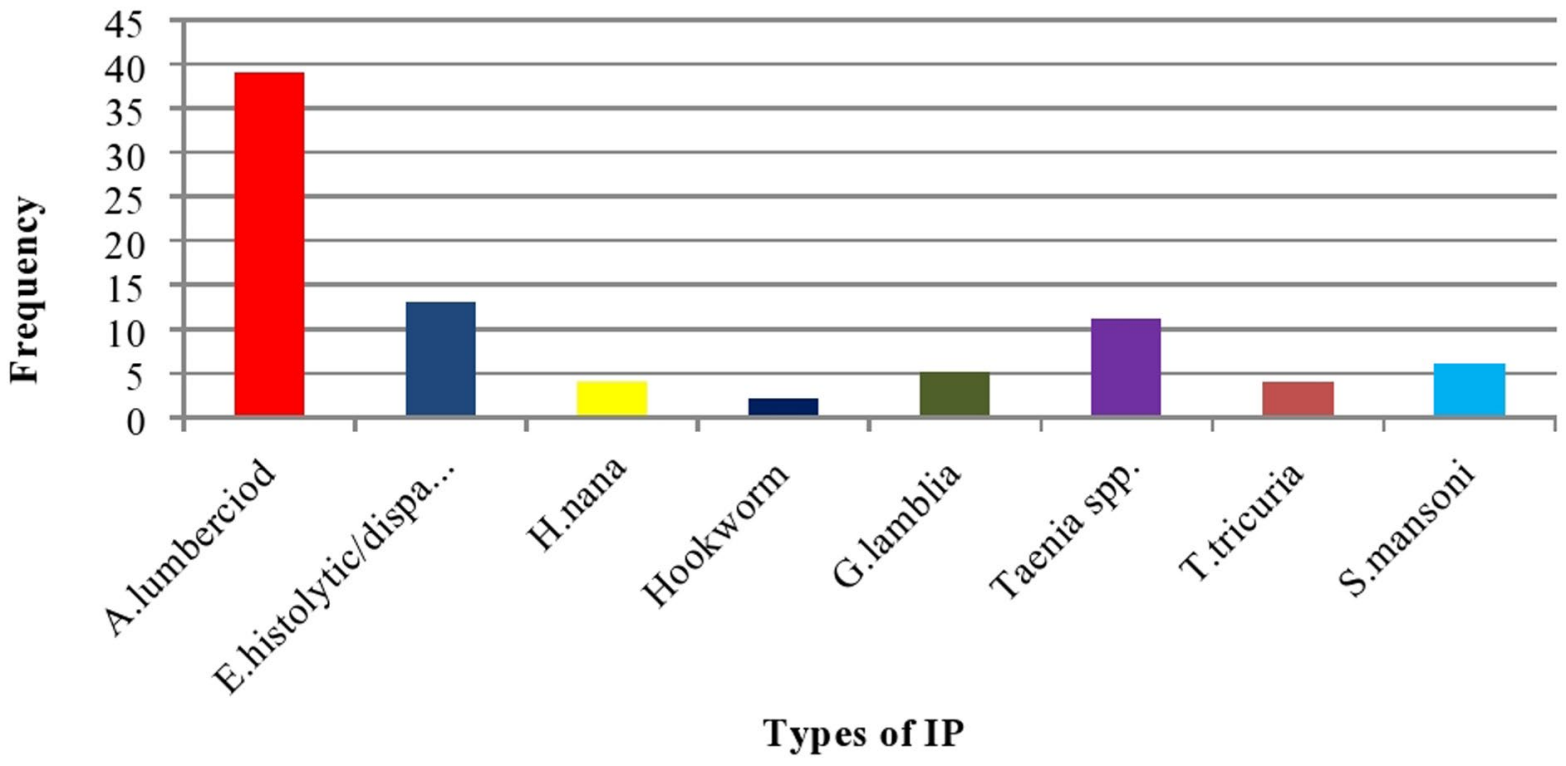
Bar graph showing the type of identified IP among postnatal women in Gondar town, Northwest Ethiopia, 2021 (*n* = 282).

The median adjusted Hb value of the women was 12.1 gm/dl (IQR: 10.8–13.1 gm/dl). A total of 126 (69.5%) women had an RBC count ≥4.0 × 10^9^/l with a mean value of 4.21 ± 0.78 × 10^9^/l. The median HCT value was 38.3% (IQR = 34.8–41.5%) with 68.4% ≥ 36%. The result of the stool examination showed that 84 (29.8%) of them were positive for at least one IP. A. *lumbercoid* was the predominant one, with a proportion of 46.4% (Table 4 and Figure 2).

**Table 4:** Laboratory findings of postnatal women in Gondar town, Northwest Ethiopia, 2021 (*n* = 282).

Laboratory tests	Categories	*n*	%
RBC count	≥4.0 × 10^9^/L	196	69.50
	<4.0 × 10^9^/L	86	30.50
HCT value	≥36%	193	68.44
	24–35.9%	77	27.30
	<24%	12	4.23
MCV	80–100 fl	259	91.84
	<80 fl	14	4.96
	>100 fl	9	3.20
MCH	<27 pg	24	8.51
	≥27 pg	258	91.49
MCHC	≥32 gm/dl	274	97.16
	<32 gm/dl	8	2.84
RDW	11–15%	207	73.40
	>15%	75	26.60
RBC morphology	Normocytic normochromic	125	94.00
	Microcytic hypochromic	5	3.75
	Macrocytic normochromic	3	2.25
Stool examination	Positive for intestinal parasite (IP)	84	29.79
	Negative for IP	198	70.21

NB; RBC, red blood cell; HCT, hematocrit; MCV, mean corpuscular volume; MCH, mean corpuscular hemoglobin; MCHC, mean corpuscular hemoglobin concentration; IP, intestinal parasite; L, liter; Pg, picogram; Fl, femtolitre; Gm/dl, gram per deciliter; RDW, red blood cell distribution width.

### Prevalence, severity, and morphologic characteristics of PPA

**Figure 3:**
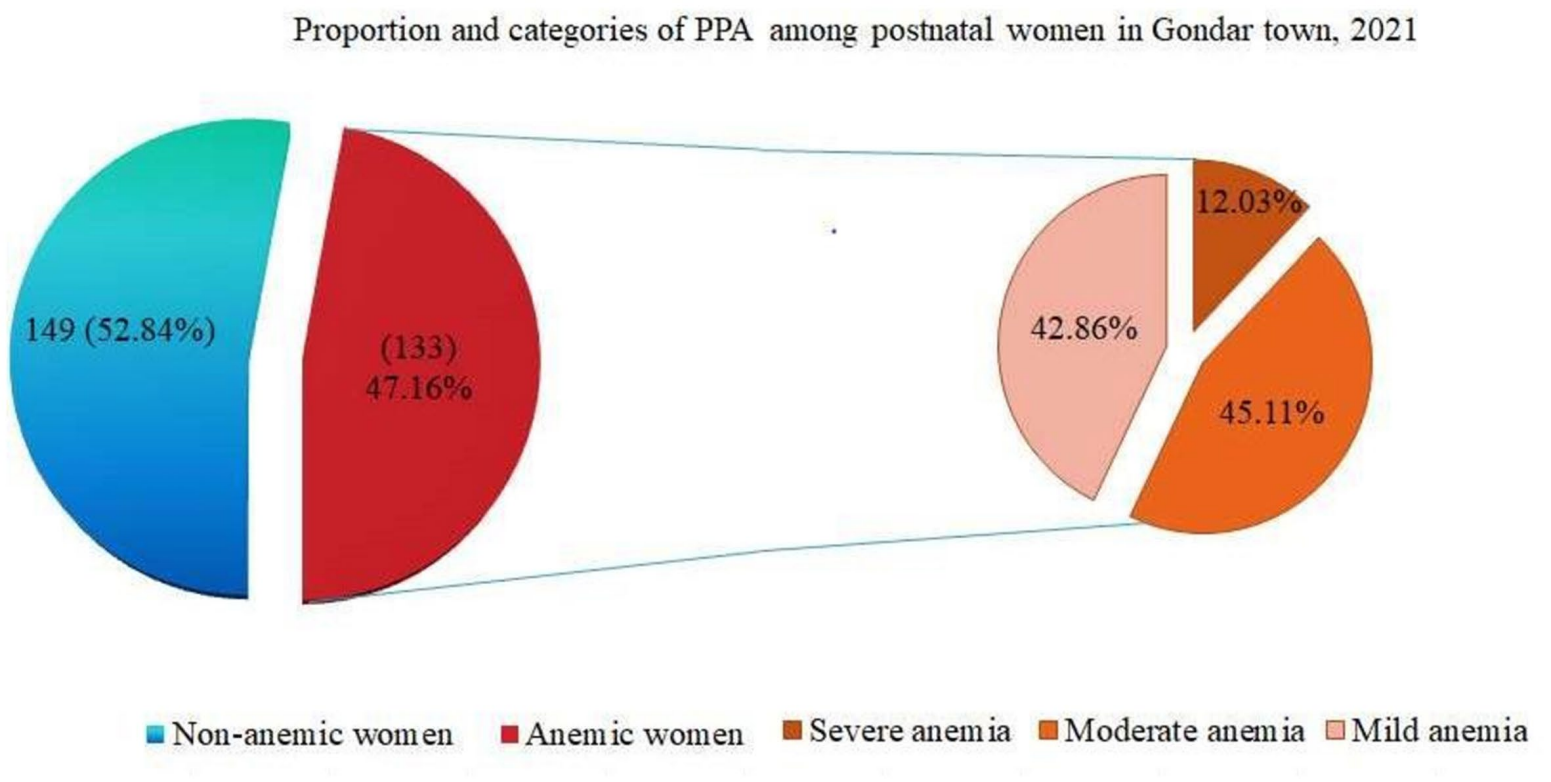
Pie of pie chart showing proportion and severity of PPA among postnatal women in Gondar town, 2021 (*n* = 282).

Prevalence and severity: The overall prevalence of PPA anemia was 133 (47.16%; 95% CI: 41.30–53.03). Individuals with anemia were further classified into three categories: mild anemia (57, or 42.86%), moderate anemia (60 or 45.11%), and severe anemia (16, or 12.03%) (Figure 3).

### Morphological characteristics of PPA

Based on the blood film reports, out of the total of 133 anemic women, the majority (125, or 94%) had a normocytic, normochromic blood picture. A total of 5 (3.76%) had a microcytic hypochromic blood picture, while 3 (2.24%) had a macrocytic blood picture (Table 4).

### Determinants of PPA in postpartum women

In univariate analysis, all variables were insignificant except PPH, mode of delivery, iron and folate supplementation during pregnancy, and level of dietary diversity. Women who experienced PPH (AOR = 2.23; 95% CI: 1.24–4.01), delivered *via* CS (AOR = 4.10; 95% CI: 2.11–7.78), did not take iron and folate supplementation during pregnancy (AOR = 2.12; 95% CI: 1.17–4.02) and had a low dietary diversity level (AOR = 1.83; 95% CI: 1.05–3.18) were at higher risk of PPA (Table 5).

**Table 5:** Logistic regression showing factors associated with PPA in Gondar town, Northwest Ethiopia, 2021 (*n* = 282).

Independent variables	Categories		PPA		COR [95% Conf. Interval]		AOR [95%Conf.Interval]
		Yes	No				
Maternal age	15–24	36	45		1.00		1.00
	25–29	56	59		1.18	(0.60–1.95)	1.42 (0.74–2.72)
	30–49	41	45		1.14 (0.63–2.10)		1.30 (0.62–2.74)
Residence	Urban	96	118		1.00		1.00
	Rural	37	31		1.47 (0.85–2.54)		0.97 (0.48–1.95)
Educational status	Unable to read and write	37	46		0.99 (0.53–1.88)		0.96 (0.46–2.04)
	Primary	38	32		1.48 (0.77–2.8)		1.02 (0.49–2.12)
	Secondary	25	30		1.04 (0.51–2.08)		1.16 (0.53–2.52)
	College and above	33	41		1.00		1.00
Marital status	Single	19	21		1. 02 (0.52–1.99)		1.05 (0.49–2.22)
	Married	114	128		1.00		1.00
Parity	Primipara	63	57		1.00		1.00
	Multiparous	70	92		0.69 (0.43–1.12)		0.48 (0.23–1.01)
PPH	Yes	95	64		3.32 (2.02–5.45)***		2.23 (1.24–4.01)**
	No	38	85		1.00		1.00
Mode of delivery	Vaginal	78	131		1.00		1.00
	CS	55	18		5.13 (2.37–9.36)***		4.10 (2.11–7.78)***
Iron and folate supplementation	Yes	89	119		1.00		1.00
	No	44	30		1.96 (1.14–3.36)**		2.12 (1.17–4.02)**
Births in 5 years	Yes	44	66		0.63 (0.39–1.01)		0.66 (0.34–1.29)
	No	89	83		1.00		1.00
ANC follow up	Yes	116	140		1.00		
	No	17	9		2.28 (0.98–5.30)		2.01 (0.69–6.24)
Tea drinking	Yes	97	94		1.60 (0.94–2.62)		1.05 (0.57–1.94)
	No	36	55		1.00		1.00
Diet diversity level	Low	83	67		2.03 (1.26–3.27)***		1.83 (1.05–3.18)*
	Minimal diet	50	82		1.00		1.00

PPA, postpartum anemia; PPH, postpartum hemorrhage; AOR, adjusted odds ratio; COR, crude odds ratio; CS, cesarean section; Conf. Interval, confidence interval. *, **, *** = *p*-values < 0.05, <0.01.

## Discussion

A number of factors have been associated with PPA. The change in Hb during the postpartum period would play an important role in providing baseline information. Thus, the current study aimed to assess the magnitude and factors associated with PPA among postnatal women in two selected health facilities in Gondar.

In this study, the prevalence of anemia among postnatal women was 47.16% (95% CI: 41.30–53.03). The finding was consistent with the results of studies conducted in southern India (47.3%) (35) and southeastern Kokang, Myanmar (56.3%) (36). In terms of severity, moderate anemia (45.11%) was common in this study. This finding was in line with a similar study conducted in India, which found 49.8% of moderate anemia types and 26% of mild anemia types (37).

On the other hand, the prevalence was lower than in studies conducted in Asia [eastern rural Myanmar (60.3%) (38) and India (76.2%; 95% CI; 70.4–81.4%) (38)]. This difference may be due to teff injera, a staple food consumed by most Ethiopian mothers (39), which contains more iron. In addition, it could be the coexistence of numerous contributing factors in rural Myanmar women like low family income, lack of primary education, hunger, drinking spring or river water, or drinking unboiled water, which could lead to anemia (38). It could be due to the fact that women in rural areas have poor socioeconomic status and, therefore, do not have access to iron-rich foods (40). Instead, given that the postpartum period is characterized by accelerated erythropoiesis and RBC mass expansion, it is possible that the hemodilution effect subsides and Hb levels return to normal in the participants in this study (35). Another reason for the discrepancy could be the difference in sample sizes, which were 227 and 733 in the India and Myanmar studies, respectively (38, 41).

The prevalence reported from the current study was higher than studies conducted in Japan (10.5%) (42), Spain, Mancha-Centro Hospital (16.4%) (22), northern Kenya (25%) (43), Ghana (16%) (44), Bhaktar, Nepal (20%) (45), Ethiopia – in Tigray (16.5%) (46), Sidama (19%) (47), Addis Ababa – at the Tikur Anbessa Specialized and Gandhi Memorial Hospitals (30%) (48), and in Debre Markos (24.3%) (30). The disparity may be due to differences in the geographical, cultural, clinical, and nutritional factors of women. In addition, different cutoff points were used to define PPA. Since there is no universally accepted definition of PPA, different researchers use different cutoff points, such as Hgb < 11 mg/dl at 24 h in Spain [Mancha-Centro Hospital (22) and in Debre Markos (30)]. In Madrid, Hgb < 10 mg/dl is used to indicate PPA for up to 6 weeks (49). In addition, there may be a difference in postpartum screening time. If the postpartum period is extended, mothers will have more time to recover from anemia or have their Hb levels increase (30). Iron is expected to improve in lactating women due to decreased iron requirements and reduced blood loss associated with amenorrhea (50).

According to RBC morphological findings, the majority of anemic women had a normocytic normochromic blood picture (94%), followed by a microcytic hypochromic (3.24%) and a macrocytic blood picture (2.24%). Possible causes include acute bleeding, or early iron deficiency (51).

Postpartum hemorrhage, iron and folate supplementation during pregnancy, CS, and low dietary diversity were found to be significantly associated with PPA. In this study, the prevalence of anemia was found to increase in women who experienced PPH. The odds of PPA were 2.2 times higher among women who had experienced PPH (AOR = 2.23; 95% CI: 1.24–4.01) than their counterparts. Probably, uterine atony, uterine inversion, coagulopathy, vaginal tears, uterine laceration, and retained tissue or placenta may expose women to cycles of bleeding, which can lead to Hb declines. The majority of studies showed Hb and HCT declines in the context of overt PPH. Excessive blood loss during and after delivery, in addition to insufficient erythropoiesis, may also cause a drop in Hb throughout the postpartum period (35).

Compared to mothers who gave birth *via* the vaginal route, mothers who gave birth *via* CS had four times higher odds of anemia (AOR = 4.10; 95% CI: 2.11–7.78). The findings were in agreement with a similar study conducted in Tigray, which discovered that vaginal birth was associated with a lower risk of anemia (AOR = 0.13; 95% CI: 0.038–0.454) (52). This potentially traumatic surgical procedure could cause women to suffer a major hemorrhage, leading to PPH. Surgery is a key determinant of blood loss. Postoperative anemia, defined as a blood loss of more than 500 ml, is a common complication that affects 80–90% of people who have had major surgery (53).

The results of this study also suggested that women who were not supplemented with iron and folate during their pregnancy had twice as many chances of developing PPA as those who were (AOR = 2.12; 95% CI: 1.17–4.02). This conclusion was supported by studies from India (AOR = 3.53; 95% CI: 1.18–11.37) and Ethiopia (35, 54). The possible explanation would be that iron is the most important nutrient for hematopoiesis and that, when taken throughout pregnancy, it has the capacity to reduce anemia, even during delivery. The depletion of stored iron during pregnancy may also be a factor because of the high demand for iron during childbirth (30).

Women with a low level of dietary diversity were 83% more likely to suffer from anemia than those with a minimum level of dietary diversity (AOR = 1.83; 95% CI: 1.05–3.18). This finding was consistent with a study conducted among lactating women in Jimma District (AOR = 2.32; 95% CI: 1.65–5.72) (24). This could be due to inadequate dietary intake, leading to an iron, vitamin B12, folate, and vitamin A deficiency. Another reason could be a lack of protein and iron-containing foods such as eggs and meat, which could lead to an iron deficiency (36).

Many studies from Ethiopia and abroad discovered that inadequate and missed ANC follow-ups, multiparity, and low education levels were independent predictors of PPA (24, 54–58). However, these factors were not associated with PPA in this study.

### Strengths and limitations

The women’s hemoglobin concentration was adjusted for altitude. To make the sampling more representative, the probability sampling technique was used. Furthermore, the study estimates were done with the appropriate statistical analysis. As a result, we are confident that this study provides more precise and generalizable results that policymakers and program managers may use to develop action strategies for this issue.

Furthermore, anemia has been associated with antepartum hemorrhage (30), but this association has not been investigated. Due to budgetary constraints, we were unable to measure anemia indicators for further categorization. As a result, we were unable to identify the type of anemia.

## Conclusion and recommendations

The prevalence of PPA in this study was a major public health concern. One in two postnatal women was found to be anemic with an adjusted Hb concentration below 12 g/dl. Iron and folate supplementation and the administration of uterotonics such as oxytocin during the third stage of labor will prevent PPH and PPA. To reduce the burden of anemia among postnatal women, health education and promotion of iron and folate supplements during pregnancy, in addition to dietary diversity practices, need to be combined with women’s long-term income-generating activities. Anemia in women who have had cesarean deliveries may also be avoided by efficient CS delivery, a positive long-term health outlook following CS, and postoperative monitoring. Thus, due attention must be given to reduce the magnitude of PPA through effective antepartum, intrapartum, and postpartum maternal care. In addition, further research is required to address the limitations of this study.

## Data availability statement

The original contributions presented in the study are included in the article/supplementary material, further inquiries can be directed to the corresponding author.

## Ethics statement

The studies involving human participants were reviewed and approved by Ethical approval and consent to participate. The research was conducted after ethical clearance was secured with reference number of SBMLS/2747 from Ethical Review Committee of School of Biomedical and Laboratory Sciences, College of Medicine and Health Sciences, University of Gondar. In addition, permission was obtained from each selected health institution. Moreover, informed consent was secured from each study participants and the obtained data were strictly confidential. Written informed consent to participate in this study was provided by the participants’ legal guardian/next of kin.

## Author contributions

GB conceived the idea, wrote the proposal, performed the data analysis and interpretation, wrote the initial draft, and revised subsequent drafts. MM and ES provided advice on study design, data analysis, and interpretation, and reviewed and commented on subsequent drafts. SK and CS participated in data collection and laboratory processing, and assisted in drafting the manuscript. MM and ES revised and edited the manuscript. All authors approved the submitted version of the manuscript.

## Funding

Funding was obtained from the University of Gondar, College of Medicine and Health Sciences.

## Conflict of interest

The authors declare that the research was conducted in the absence of any commercial or financial relationships that could be construed as a potential conflict of interest.

## Publisher’s note

All claims expressed in this article are solely those of the authors and do not necessarily represent those of their affiliated organizations, or those of the publisher, the editors and the reviewers. Any product that may be evaluated in this article, or claim that may be made by its manufacturer, is not guaranteed or endorsed by the publisher.

## Abbreviations

ANC: Antenatal Care
AOR: Adjusted Odds Ratio
BMI: Body Mass Index
CI: Confidence Interval
COR: Crude Odds Ratio
CS: Cesarean Section
DDS: Dietary Diversity Score
FAO: Food and Agricultural Organization
G/dl: gram per deciliter
Hb: hemoglobin
HCT: hematocrit
HIV: human immunodeficiency virus
IDA: iron deficiency anemia
IP: intestinal parasite
IQR: interquartile range
K3-EDTA: tri-potassium ethylene diamine tetra-acetic acid
KM: kilometer
MCH: mean corpuscular hemoglobin
MCHC: mean corpuscular hemoglobin concentration
MCV: mean corpuscular volume
Ml: milliliter
PNC: postnatal care
PPA: postpartum anemia
PPH: postpartum hemorrhage
QC: quality control
RBC: red blood cell
RDW: red cell distribution width
RPM: revolutions’ per minute
UGCSH: University of Gondar Comprehensive Specialized Hospital
WHO: World Health Organization
